# Matrix Metalloproteinase-3 (MMP-3) Polymorphisms Are Associated with Prolonged ECG-Derived QTc Interval: A Cross-Sectional Study of the Australian Rural Population

**DOI:** 10.3390/jpm11080705

**Published:** 2021-07-23

**Authors:** Yaxin Lu, Nathan Ussher, Yuling Zhou, Herbert Jelinek, Brett Hambly, Amy Li, Craig S. McLachlan

**Affiliations:** 1 JL Operating Theatres, Royal Prince Alfred Hospital, Camperdown 2050, Australia; Yaxin.Lu@health.nsw.gov.au; 2 Rural Clinical School, University of New South Wales, Sydney 2052, Australia; nathanussher@gmail.com; 3 Xiamen Cardiovascular Hospital, Xiamen University, Xiamen 361005, China; zhouyuling@xmheart.com; 4 The School of Economics, Xiamen University, Xiamen 361005, China; 5 Health Sciences, Charles Sturt University, Albury 2640, Australia; hjelinek@csu.edu.au; 6 Department of Pathology, University of Sydney, Sydney 2006, Australia; brett.hambly@laureate.edu.au; 7 Center for Healthy Futures, Torrens University, Pyrmont 2009, Australia; craig.mclachlan@torrens.edu.au; 8 Department of Pharmacy & Biomedical Sciences, La Trobe University, Flora Hill 3552, Australia

**Keywords:** matrix metalloproteinases, MMP-3, rural populations, ECG

## Abstract

Matrix metalloproteinases (MMPs) are enzymes that are integral in extracellular matrix (ECM) remodeling. In age or disease, ECM may become dysregulated and contribute to fibrosis, which impairs cardiac electrical conduction. Two alleles regulate matrix metalloproteinase-3 (MMP-3) activity: one with five adenosine bases (5A; associated with higher MMP-3 activity and decreased fibrosis) and another with six adenosine bases (6A; associated with lower MMP-3 activity and increased fibrosis). Here, we determined whether ECG-derived QTc and related parameters are associated with the MMP-3 5A/6A genotype in a cross-section of the Australian rural population. A retrospective cross-sectional population was obtained from the Charles Sturt University Diabetes Screening Research Initiative. Genotype and resting 12-lead ECG parameters of 295 participants were analyzed. Amongst these participants, 85 individuals carried the 5A/5A genotype, 141 individuals carried the 5A/6A genotype, and 65 individuals carried the 6A/6A genotype. Compared to 5A/5A genotype carriers, 5A/6A genotype carriers had a significantly longer QTc duration by 9.50 ms (95% CI: 3.48–15.52, *p* = 0.002), whilst 6A/6A genotype carriers had an even longer QTc duration by 12.19 ms (95% CI: 5.04–19.34, *p* = 0.001). We found an association between MMP-3 5A/6A polymorphisms and QTc, independent of adjustments for age, gender, alcohol consumption, smoking status, body mass index and blood pressure.

## 1. Introduction

Matrix metalloproteinases (MMPs) are a family of zinc-dependent endopeptidases that are integral to the normal and pathological remodeling of the cardiac extracellular matrix (ECM). The ECM is a connective tissue scaffold that provides structural and functional support to cells in the myocardium and is pivotal in maintaining the excitability of the cardiac electrical conduction system [1]. Disruption to the normal balance of ECM degradation and synthesis mediated by MMPs results in corresponding dilatation and fibrosis that were previously associated with age-related conduction disorders [1,2] along with the development of cardiovascular diseases [3,4], including atrial fibrillation [5].

MMP-3 (stromelysin-1), secreted by cardiac fibroblasts and macrophages, plays a key role in proteolytically digesting specific components of the ECM and can also activate a number of other MMPs [6]. A common functional 5A/6A polymorphism in the promoter region of the MMP-3 gene, which has a run of five adenosines (5A) on one allele and six adenosines (6A) on the second allele at nucleotide −1171, influences MMP-3 promoter activity and transcription factor binding [3]. This 5A/6A polymorphism has been extensively reported on in gene association studies with links to myocardial infarction, angina, ischemic stroke, deep venous thrombosis, abdominal aortic aneurysm, coronary artery stenosis, hypertension and arrhythmias [7,8,9,10,11].

Polymorphisms in the MMP-3 gene translate into measurable differences in phenotypic outcome. In vitro gene reporter assays demonstrated that the 5A allelic promoter activity was approximately two-fold higher than the 6A allelic promoter activity in driving MMP-3 gene expression [12]. In agreement, an in vivo study confirmed that the 5A allele is associated with increased MMP-3 transcription, but only in specific environmental conditions involving inflammation [13]. Further studies also reveal associations between MMP-3 5A/6A polymorphisms, atherosclerosis and vascular remodeling in both humans and animals [3,14]. These data imply that the 5A allele (i.e., 5A/5A or 5A/6A genotype) with its higher MMP-3 activity would favor ECM degradation that has been associated with acute coronary events [15] and aortic aneurysms [8]. In contrast, it was postulated that the 6A allele, with reduced MMP-3 activity, tends to accumulate ECM and is associated with coronary stenosis [14] and the formation of ECM-rich stable atherosclerotic plaques [3,12]. Thus, 6A/6A genotypes are associated with phenotypes of ECM deposition, whereas 5A/6A and 5A/5A genotypes are associated with phenotypes involving ECM degradation. Surprisingly, both alleles are involved in remodeling that results in adverse cardiovascular outcomes, indicating that MMP-3 homeostasis is essential to normal function. 

Since MMP-3 is an active player in ECM homeostasis, we hypothesize that 5A and 6A polymorphisms associated with adverse myocardial remodeling, which increases collagen synthesis and disrupts efficient electrical conduction, may be associated with electrocardiogram (ECGs) changes. Few studies have investigated the interaction of MMP-3 genotypes with differential ECG parameters. Our aim is to explore the association of MMP-3 5A/6A polymorphisms on ECG parameters in an ageing rural Australian population. These models will be adjusted for conventional cardiovascular disease risk factors (e.g., smoking, hypertension, and diabetes). 

## 2. Materials and Methods

### 2.1. Study Population and Design

This retrospective study was based on biobanked blood samples from a community health screening project conducted at the Albury-Wodonga campus of Charles Sturt University, Australia. For this study, 295 participants with complete clinical phenotypic data, ECG data and whole blood samples were selected. Written informed consent was obtained from each subject. Ethical approval for the study was obtained from the Charles Sturt University Human Research Ethics Committee (protocol number 2006/042). 

### 2.2. Data Collection

A standardized questionnaire was used to collect demographic information such as age, sex, smoking history, alcohol consumption, height, and weight (to calculate body mass index). Clinical histories were also collected as part of the screening, which documented pre-existing medical conditions such as hypertension, cardiovascular disease, and diabetes mellitus. Subjects were classified as “consuming alcohol” if they answered “yes” to drinking more than two to three standard drinks per day.

### 2.3. Blood Collection and Biochemistry Assessment

Venous blood samples were collected in EDTA-containing tubes. Fasting glucose levels, glycated hemoglobin (HbA1C), low-density lipoprotein (LDL), high-density lipoprotein (HDL), total cholesterol, and triglycerides were measured. All participants were asked to fast for a minimum of 8 h prior to attending the health screening. These samples were processed at South West Pathology, Albury. 

### 2.4. Blood Pressure Measurements

Participants rested for a 5 min period prior to blood pressure measurements. Blood pressure was measured with a standard mercury sphygmomanometer (Welch Allyn Australia P/L) in a supine position. The blood pressure cuff was placed on the upper arm and measurements were taken then repeated one minute later and the average was recorded.

### 2.5. 12-Lead Electrocardiogram (ECG)

Resting 12-lead ECGs were performed using a Welch Allyn PC-based ECG that automatically calculated the following ECG parameters from a 10 s ECG strip: PQ interval (PQi), QRS duration (QRSd), QT dispersion (QTd), QT interval (QTi), and the corrected QT interval (QTc). The QTi parameter represents the duration between the onset of ventricular depolarization and completion of repolarization. However, QTi measurements will vary depending on age and gender and are inversely proportional to heart rate [16]. In clinical practice, QTi is often adjusted for heart rate (HR) resulting in a corrected QTi (QTc) using the Bazett formula (see below), where RR is the time between two successive R waves. 

**Bazett’s correction for QT interval (**$QT_{c}$**)**:

$${\mathrm{QT}}_{c}=\frac{{\mathrm{QT}}_{i}}{\sqrt{\mathrm{RR}}}=\frac{{\mathrm{QT}}_{i}}{\sqrt{\frac{60}{\mathrm{HR}}}}={\mathrm{QT}}_{i}\sqrt{\frac{\mathrm{HR}}{60}}$$

### 2.6. DNA Genotyping

DNA from whole blood samples was extracted in line with the manufacturer’s instructions (QIAamp DNA Mini Kit; QIAamp^®^, Qiagen, Chadstone, VIC, Australia). MMP-3 genotypes were then determined using pyrosequencing. To differentiate between (5A/5A, 6A/6A) homozygous and (5A/6A) heterozygous genotypes, specific primers that would anneal immediately adjacent to the single nucleotide polymorphisms (SNP) of interest were designed using PyroMark assay design software. Two single-stranded DNA fragments (SigmaTM), identical to the sequences of the 118 bp PCR fragment containing 5A in the SNP region and the 119 bp PCR fragment containing 6A in the SNP region, were used as positive controls to validate the pyrosequencing results. A self-priming oligonucleotide was also used to validate the viability of the vacuum workstation and the performance of the PyroMark Q24 instrument. To reveal any contamination in the reagents used or non-specific products formed from the PCR reactions, several negative controls were used. Assays were run without the sequencing primer, without the ssDNA-5A and -6A, and without patient DNA to check for contamination in their corresponding solutions.

### 2.7. Statistical Analysis

Data were expressed as means and standard deviation (SD). Uni-variable and multivariable general linear models were applied to analyze the relationship between blood pressure, ECG parameters and MMP-3 5A/6A genotypes. Possible associations between MMP-3 5A/6A polymorphisms and cardiovascular risk factors (diabetes mellitus, cardiovascular disease, and hypertension) were investigated using multivariable logistic regression models. All data were analyzed using SPSS 24.0 (IBM, Armonk, New York, NY, USA). A two-sided *p*-value < 0.05 was considered statistically significant.

## 3. Results

This study population of 295 subjects older than 25 years was obtained from the rural Australian community with an average age of 64.88 ± 10.17 years, with 42.7% of the cohort being male (Table 1). In this study population, 94 participants were treated for diabetes mellitus with one prediabetic and 89 of those having the type 2 variant; the remaining 200 participants did not have diabetes mellitus. In this cohort, 65 participants had cardiovascular disease (22.0%), 162 participants had hypertension (54.9%) consistent with the above average (>130 mmHg) SBP values, six participants were current smokers (2.0%), and 24 participants reported consuming more than two to three glasses of alcohol per day (8.1%).

Associations between MMP-3 polymorphism and blood pressure (SBP, DBP) or ECG parameters (PQi, QRSd, QTc, QTd) are presented in Table 2. SBP and DBP were not associated with 5A/6A or 6A/6A polymorphisms. The PQi, QRSd, and QTd measurements revealed no significant differences (*p* = NS) between the polymorphisms. In contrast, QTc duration was prolonged in carriers of the 5A/6A (*p* = 0.002) and 6A/6A (*p* = 0.002) genotype in comparison to the 5A/5A genotype. The average QTc durations of the 5A/5A, 5A/6A, and 6A/6A genotypes were 422.08 ± 19.09 ms, 431.91 ± 22.92 ms, and 433.39 ± 25.82 ms, respectively. 

Factors that that may have influenced QTc duration were adjusted for in the multivariate models (Table 3). Covariates adjusted included age, gender, alcohol status, smoking status, BMI, SBP, and DBP. We confirm that the QTc of the 5A/6A genotype carriers was longer by 9.50 ms (95% CI: 3.48–15.52, *p* = 0.002) and for 6A/6A genotypes by 12.19 ms (95% CI: 5.04–19.34, *p* = 0.001) compared to 5A/5A genotype carriers. 

Further exploratory analysis for cardiovascular risk factors and MMP3 polymorphisms adjusted by age, gender, alcohol consumption, smoking status, and BMI is detailed in Table 4. The 5A/6A and 6A/6A genotype showed no significant associations with diabetes mellitus (*p* = NS), cardiovascular disease (*p* = NS), or hypertension (*p* = NS). 

## 4. Discussion

This study examined associations between MMP-3 5A/6A polymorphisms and ECG-derived cardiac conduction parameters in a cross-sectional Australian rural population. We found that QTc interval was associated with MMP-3 5A/6A and 6A/6A polymorphisms. Prolonged QTc interval persisted in individuals carrying the 6A allele independent of adjustments for age, gender, alcohol consumption, smoking status, BMI, and blood pressure. It should be noted that QTc prolongation as described in this study (431.91 ± 22.92 ms for 5A/6A and 433.39 ± 25.82 ms for 6A/6A) remains within the physiological range and should not be confused for long QT syndrome (QTc > 500 ms) [17,18].

Several studies had reported that QTc prolongation was strongly associated with age [19,20,21]. The highest incidence of prolonged QTc was identified in individuals aged over 80 years but decreased in those under the age of 50 years [20]. It is therefore unsurprising that we found no further age-adjusted differences as our study cohort consists of participants with a mean age of 64.88 ± 10.17 years, falling well within the reported age brackets with the least (<50 years old) and greatest (>80 years old) effects on QTc. Notably, Zhang et al. (2011) found that a 50 ms prolongation of QTc duration increased the relative risk of total mortality by 1.20, cardiovascular mortality by 1.29, coronary heart disease mortality by 1.49 and sudden cardiac death by 1.24 [22]. This metanalysis suggests that the 5A/6A and 6A/6A genotypes, which further prolong QTc in addition to age, are a factor that may increase the risk of acquiring a cardiovascular disease. 

While there are several possible factors that influence QTc, we identified that MMP-3 polymorphisms may in part contribute to ECM remodeling in older populations and be reflected as a prolonged QTc interval. In our cohort of older rural Australians, 71% carried the 5A/6A or 6A/6A genotype. Since MMP-3 has been implicated in degrading specific components of the ECM [23], the 6A allele that is associated with reduced MMP-3 promoter activity may increase matrix deposition in the heart, resulting in fibrosis [12] that accompanies physiological ageing [24]. The MMP-3 6A allele may also influence the creation of sparsely interconnected strands of myocytes that become electrically isolated from one another by collagen bundles. Indeed, fibrosis can force electrical impulses to form a zigzag pathway through the tissue, delaying cardiac propagation of electrical impulses [25]. Our findings suggest that the 6A allele may physiologically influence the extended duration between ventricular depolarization and repolarization. It is appreciated that an already extended QTc interval of older individuals carrying the 6A allele would be more susceptible to reaching clinically relevant QTc in the presence of common medications known to prolong QTc [23].

Additionally, the coordinated action of cardiac myocytes is created by gap junctions, which allow electrical coupling and the direct cell–cell transfer of chemical signals between cells. The building blocks of these gap junctions are various connexin (Cx) proteins, which are differentially expressed in the cardiac chambers and conduction system. For instance, Cx40 and Cx43 co-expressed in the atria, while Cx43 is the only connexin found within the ventricular myocardium [26]. Not only do MMPs remodel the extracellular matrix, but they also cleave intracellular proteins such as connexins [27]. In fact, the differential expression of connexins between the atria, ventricular and conduction system is thought, in part, to be due to MMPs. For instance, in cardiac fibroblasts, increased expression of MMP-2 and MMP-9 has been associated with concomitant decreases in Cx43 expression [28]. Reduced expression of Cx43 has been shown to increase fibrosis in stressed mouse hearts, impeding linear conduction [29]. Given the role of MMP-3 as an upstream activator of many other MMPs, including MMP-1, -7, and -9 [4], which in turn alter Cx protein expression, this may help explain the differences in the QTc between MMP-3 genotypes noted in our study.

Furthermore, our study found no association between MMP-3 polymorphisms and QRS duration. In contrast, one previous study investigated the associations between MMP-3 5A/6A polymorphisms and ECG-derived QRS duration [30]. Unsurprisingly, this study examined 184 chronic heart failure patients stratified into ischemic and non-ischemic causes. Despite no correlation between MMP-3 5A/6A polymorphisms and ventricular remodeling, subjects with the MMP-3 6A allele had increased QRS duration. Correspondingly, subjects with the 5A/5A genotype and a non-ischemic cause of heart failure appeared protected from significant changes in QRS parameters. Intriguingly, increased MMP-3 protein expression was also previously identified in non-ischemic but not in ischemic dilated cardiomyopathy patients [31]. Together, these data suggest that MMP-3 may contribute to ventricular remodeling in specific forms of heart failure, resulting in impaired electrical conductivity. Our study, to the best of our knowledge, is the first to examine potential associations with MMP-3 5A/6A polymorphisms and cardiac conduction in a cohort of patients without heart failure.

Nevertheless, our results should be interpreted in the context of certain limitations. We used the autogenerated QTc formula from the Welch Allyn PC system to calculate QTc values. QTc is approximately normally distributed in the general population and normal ranges have been stratified according to age (i.e., children versus adults) and gender. Whilst there has been some debate about this, in 2009, a joint report from the American Hospital Association, American College of Cardiology, and other professional organizations recommended that the normal range of QTc in women be set at 390–460 ms, while the normal range of QTc for men be set at 390–450 ms [18]. However, a recent metanalysis has shown that 10–20% of otherwise healthy individuals may have QTc values outside this range [22]. Similarly, while many authors consider values greater than 500 ms as a cause for concern, QTc values greater than 500 ms have certainly been recorded in healthy individuals [16]. Whilst it has been contentious which cut-off value to use for the diagnosis of an abnormal QTc, we elected to use continuous values of QTc in our regression models. The utility of various QTc formulas and automated screening tools have been reported on in several studies [32,33,34]. While there is no consensus on the best formula to be used in clinical practice, in resting conditions with heart rates in the 60–90 beats/min range, these formulae generally provide equivalent results when diagnosing QT prolongation [35] and so should not significantly affect our results. 

## 5. Conclusions

In summary, we observed an association between MMP-3 5A/6A polymorphisms and QTc interval in a rural aging population. The 5A/6A and 6A/6A genotypes possibly stimulate subtle changes in the myocardial ECM structure that is associated with increased matrix deposition—enough to influence cardiac conduction in the ageing population largely free of overt cardiovascular diseases. Clinicians treating carriers of the 6A genotype who are seemly predisposed to longer QTc durations than their 5A/5A counterparts should take the phenotype into consideration when prescribing treatments that further prolong QTc duration and may lead to electrical conduction disorders such as torsade de pontes.

## Figures and Tables

**Table 1:** Baseline characteristics of the Australian rural population. Demographic, blood biochemistry, blood pressure, and ECG values.

Factor	Mean ± SD	N (%)
N		295
Age (years)	64.88 ± 10.17	-
Sex		
Female (%)		169 (57.3)
Male (%)		126 (42.7)
BMI (kg/m^2^)	28.42 ± 4.93	
Diabetes mellitus		
Prediabetes (%)		1 (0.3)
Type I (%)		5 (1.7)
Type II (%)		89 (31.2)
Cardiovascular disease (%)		65 (22.0)
Hypertension (%)		162 (54.9)
Smoking (%)		6 (2.0)
Alcohol (%)		24 (8.1)
SBP (mmHg)	130.19 ± 17.42	
DBP (mmHg)	76.61 ± 9.81	
Glucose (mM)	6.04 ± 2.57	
HbA_1c_ (%)	6.11 ± 1.13	
TC (mM)	5.00 ± 1.17	
triglyceride (mM)	1.41 ± 0.80	
HDLc (mM)	1.37 ± 0.38	
LDLc (mM)	3.00 ± 1.04	
TC/HDL	3.82 ± 1.09	
PQ_i_ (ms)	174.58 ± 28.24	
QRS_d_ (ms)	101.54 ± 17.65	
QT_c_ (ms)	429.42 ± 23.04	
QT_d_ (ms)	62.77 ± 32.43	

BMI, body mass index; SBP, systolic blood pressure; DBP, diastolic blood pressure; HbAc1, glycated hemoglobin; TC, total cholesterol; HDLc, high-density lipoprotein cholesterol; LDLc, low-density lipoprotein cholesterol; CVD, cardiovascular disease.

**Table 2:** Blood pressure and ECG parameters across MMP-3 polymorphisms.

	5A/5A (*n* = 85)	5A/6A (*n* = 141)			6A/6A (*n* = 69)		
		Coefficient *	95% CI	p	Coefficient *	95% CI	p
SBP	Reference ^^^	0.21	(−4.51, 4.94)	0.930	−0.46	(−6.04, 5.11)	0.871
DBP	Reference ^^^	−0.14	(−2.79, 2.51)	0.918	1.79	(−1.34, 4.91)	0.262
PQ	Reference ^^^	−3.82	(−11.52, 3.86)	0.328	−8.10	(−17.18, 0.99)	0.081
QRS	Reference ^^^	2.51	(−2.26, 7.28)	0.301	3.53	(−2.11, 9.16)	0.219
QTc	Reference ^^^	9.83	(3.71, 15.94)	0.002	11.31	(4.09, 18.53)	0.002
QTd	Reference ^^^	−0.68	(−10.01, 8.66)	0.887	0.94	(−10.03, 11.91)	0.866

^ wild type allele or genotype served as a reference; * calculated using general regression model, without adjustment.

**Table 3:** Multivariate logistic regression analysis between ECG parameters and MMP-3 polymorphisms.

	5A/5A	5A/6A			6A/6A		
		Coefficient *	95%CI	p	Coefficient *	95%CI	p
PQ	Reference ^^^	−3.74	(−11.14, 3.65)	0.320	−8.33	(−17.14, 0.47)	0.063
QRS	Reference ^^^	2.54	(−2.01, 7.09)	0.272	3.033	(−2.37, 8.44)	0.270
QTc	Reference ^^^	9.50	(3.48, 15.52)	0.002	12.19	(5.04, 19.34)	0.001
QTd	Reference ^^^	−0.50	(−9.87, 8.87)	0.916	1.64	(−9.45, 12.72)	0.771

^ wild type allele or genotype served as a reference; * calculated using general regression model, adjusted by age, gender, alcohol status, smoking status, BMI, SBP, and DBP.

**Table 4:** Multivariate logistic regression analysis between cardiovascular risk factors and of MMP-3 polymorphisms.

	5A/5A	5A/6A			6A/6A		
		OR *	95%CI	p	OR *	95%CI	p
Diabetes mellitus	1.00	1.00	(0.55, 1.52)	0.999	1.23	(0.61, 2.48)	0.566
CVD	1.00	1.54	(0.76, 3.13)	0.234	1.04	(0.44, 2.45)	0.934
Hypertension	1.00	0.74	(0.41, 1.35)	0.331	1.03	(0.50, 2.12)	0.928

* calculated using logistic regression model, adjusted by age, gender, alcohol consumption, smoking status, and BMI.

## Data Availability

The data presented in this study may be available upon reasonable request from the authors (A.L., C.S.M.). The data are not made publicly available due to ethical considerations as it is linked to clinical data.
